# The Development of Integrin Alpha-8 Deficient Lungs Shows Reduced and Altered Branching and a Correction of the Phenotype During Alveolarization

**DOI:** 10.3389/fphys.2020.530635

**Published:** 2020-12-21

**Authors:** Tiziana P. Cremona, Andrea Hartner, Johannes C. Schittny

**Affiliations:** ^1^Institute of Anatomy, Department of Preclinical Medicine, Faculty of Medicine, University of Bern, Bern, Switzerland; ^2^Department of Pediatrics and Adolescent Medicine, University Hospital of Erlangen-Nürnberg, Erlangen, Germany

**Keywords:** integrin alpha 8, lung development, branching morphology, alveolarization, transgenic mice, integrin

## Abstract

Lung development involves epithelial–mesenchymal interactions and integrins represent one of the key elements. These extracellular matrix receptors form hetero-dimers of alpha and beta subunits. The integrin α8β1 is highly expressed in mouse tissues, including lung. It forms a cellular receptor for fibronectin, vitronectin, osteopontin, nephronectin, and tenascin-C. This study aims to investigate the role of the integrin α8-subunit (α8) during lung development. Wild type and α8-deficient lungs were explanted at embryonic days 11.5/12.5. After 24–73 h in culture α8-deficient lung explants displayed reduced growth, reduced branching, enlarged endbuds, altered branching patterns, and faster spontaneous contractions of the airways as compared to wild type. Postnatally, a stereological investigation revealed that lung volume, alveolar surface area, and the length of the free septal edge were significantly reduced in α8-deficient lungs at postnatal days P4 and P7. An increased formation of new septa in α8-deficient lungs rescued the phenotype. At day P90 α8-deficient lungs were comparable to wild type. We conclude that α8β1 takes not only part in the control of branching, but also possesses a morphogenic effect on the pattern and size of the future airways. Furthermore, we conclude that the phenotype observed at day P4 is caused by reduced branching and is rescued by a pronounced formation of the new septa throughout alveolarization. More studies are needed to understand the mechanism responsible for the formation of new septa in the absence of α8β1 in order to be of potential therapeutic benefit for patients suffering from structural lung diseases.

## Introduction

### Lung Development

The lungs are the main organ of respiration present in human, mice, and higher vertebrates. They provide a large internal surface area where the inspired air and the capillary blood get in close contact to each other to allow an efficient exchange of gases. To achieve this goal, during lung development, six important and overlapping stages occur: organogenesis, pseudoglandular stage, canalicular stage, saccular stage, alveolarization, and microvascular maturation. Successful development and function of the lung also involves the biochemical development of the surfactant system required for the stability and immune defense of the large respiratory surface area (Schittny, 2017).

During the pseudoglandular and canalicular stage, large parts of the pulmonary airways are formed prenatally by branching morphogenesis. In humans, monkeys, dogs and others, the bronchial tree is formed by a repetitive dichotomous branching of the future airways whereas in rodents, branching follows a monopodial pattern (Woods and Schittny, 2016).

Mostly postnatally in rodents and human the gas exchange surface of the lungs is enlarged during alveolarization; new septa are lifted off of pre-existing ones subdividing the most distal airspaces (Mund et al., 2008; Schittny et al., 2008). Alveolarization is a process, which does not finish during early childhood but continues until young adulthood (for a review see Schittny, 2017, 2018). Throughout lung postnatal development of rodents the size of the alveoli first decreases and later increases (Mund et al., 2008; Tschanz et al., 2014). Therefore, the alveolar surface area, the number of alveoli, and the total length of free septal edge does not increase in parallel with the increase of the lung volume.

### Integrins

Cellular and extracellular signaling networks orchestrate lung development. In the past the extracellular matrix was mainly viewed as the structural element of tissue. In the last two decades it became obvious that the extracellular matrix represents a dynamic and functional active zone that contributes to the establishment of cellular phenotypes. The contact to specific extracellular matrix proteins precisely modulates differentiation, migration, proliferation, and programmed cell death (Streuli, 1999). Extracellular matrix proteins like elastin, collagens, laminins and tenascin-C contribute to pre and postnatal development. These proteins are recognized by specific receptors such as integrins, α-dystroglycan and other collagen receptors (Arnaout et al., 2005). Integrins are a large family of heterodimeric transmembrane glycoproteins containing a single α and a single β subunit non-covalently linked. The expression, distribution, and role of the different integrins present on airway epithelial cells have been characterized *in vivo* and *in vitro* (Coraux et al., 1998; Sheppard, 2003).

### α8β1 Integrin

The integrin α8 subunit exclusively associates with the integrin β1 subunit to form receptors for fibronectin, vitronectin, nephronectin, osteopontin, and tenascin-C (Muller et al., 1995; Schnapp et al., 1995a, b; Brandenberger et al., 2001). In the lung the presence of α8β1 appears prenatally diffusely in the mesenchyme except for cells surrounding the branching and growing ends of the future airways. Postnatally, α8 keeps its diffuse mesenchymal distribution, but co-localizes with fibronectin and also with alpha smooth muscle actin (Chiquet-Ehrismann and Tucker, 2011). It has been shown that integrin α8β1 mediates signals from the extracellular matrix to alveolar myofibroblasts and contributes to secondary septation (Wagner et al., 2003).

### α8β1 Integrin Knock Out Mice

In 1997 Müller and coworkers created an integrin α8-deficient mouse line by inactivating the gene encoding the α8 subunit by gene targeting (Muller et al., 1997). They demonstrated that α8β1 plays a crucial role in epithelial–mesenchymal interactions during kidney morphogenesis. This explains renal agenesis or dysgenesis at birth in homozygote mice. The penetration of this phenotype varies between two normal kidneys and no kidneys at all. Further investigations were necessary to discover that they have inner ear defects (Littlewood and Muller, 2000), and in the lung, anomalies in airway division and fusion of the medial and caudal lung lobes (Benjamin et al., 2009). The spatiotemporal expression pattern in lung development has been studied (Wagner et al., 2003) but detailed morphometric studies during prenatal and postnatal lung development have never been done.

### Aim of the Study

The aim of this study is to investigate the role of the integrin α8 subunit during lung development. We hypothesized that the α8 subunit plays an essential role in two important mechanisms: branching morphogenesis and alveolarization. To test these hypotheses, we have performed lung organ culture and morphometric studies during prenatal and postnatal lung development. We observed reduced branching, an altered branching pattern, and a higher frequency of spontaneous contractions of the future airways during prenatal development of α8 deficient lungs resulting in a reduced lung volume, surface area, and length of the free septal edge at postnatal day 4. A tremendous amount of septa are formed to rescue the prenatal phenotype of α8-deficient mice until adulthood. We conclude that integrin α8β1 takes part in the control of branching morphogenesis and that during alveolarization an incompletely developed lung may be rescued by an increased alveolarization.

## Materials and Methods

### Animals

The α8-deficient mouse strain, kindly gift from Dr. Müller, was used (Muller et al., 1997). Wild type (WT) 129/Sv mice were used as age matched controls. 12.5 days old fetuses were obtained from heterozygote and the 11.5 days old ones from homozygote breeding (Table 1). Postnatal lungs were obtained from homozygote breading. All animal studies were approved by and conducted in accordance with the Veterinary Service of the Canton of Bern and the Canton of Basel, Switzerland and the Swiss Federal Agency for Environment, Forest and Landscape.

**TABLE 1:** Summary of lung organ experiments.

	Number of explants			Penetration of branching phenotype
	KO+	KO−	WT	
Experiment 1 (E12.5)	6	3	20	66%
Experiment 2 (E11.5)	5	2	3	57%
Experiment 3 (E11.5)	6	2	4	75%
Total	17	7	27	71%

*The number of used explants and the penetration of phenotype is given. KO+, explants with reduced branching, KO−, explants showing similar numbers of branches as the wild type (WT) explants.*

### Lung Organ Culture

Fetal mouse lungs were obtained at day E11.5 or E12.5 under sterile conditions and cultured as described previously (Schittny et al., 2000; Roth-Kleiner et al., 2004). Depending on the size of the lungs they were either explanted as entire lungs (E11.5) or their lobes were separated (E12.5) in sterile phosphate buffered saline (127 mM NaCl, 10 mM Na_2_HPO_4_, pH 7.4). In both cases, the explants were cultured individually at the air-culture medium interface on a floating filter (TSTP Isopore filter, 13 mm diameter, 3 μm pore size; Millipore, Bedford, MA, United States) in a 24 well plate (Falcon-Becton Dickinson, Lincoln Park, NJ, United States) at 37°C in 5% CO_2_/air. Lungs obtained at E11.5 were explanted undissected. The medium, Dulbecco’s modified eagle medium(GibcoBRL-Life Technologies, Basel, Switzerland), was enriched with 10% fetal calf serum (GibcoBRL), 5 μg/ml bovine insulin (Sigma Chemicals Company, St. Louis, MO, United States), 2 mM glutamine (Sigma), 75 μg/ml Streptomycin (Sigma), and 100 μg/ml Penicillin (Sigma). Three explants were transferred onto each filter and the filters were placed in a well containing 350 μl medium. The distance between the three explants was large enough to ensure the tissues did not touch during growth. The explants were fed daily by adding 200 μl of fresh medium per well. Images of the explants were taken twice daily using an inverted microscope (Diaphot-TMD, Nikon, Tokyo, Japan).

### Quantitative Evaluations of Organ Cultures

**FIGURE 1:**
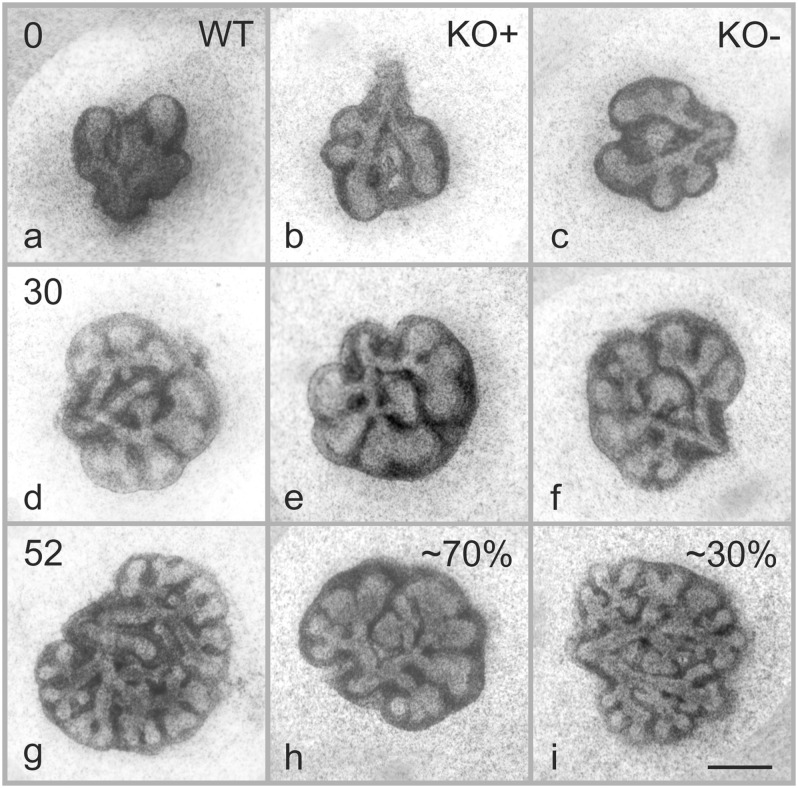
Effects of α8 deficiency on branching morphogenesis. Representative micrographs of lung organ culture from WT and α8 deficient (KO) lung explants. Images were taken at the start of the organ culture E11.5 (**a–c**, time 0), 30 **(d–f)**, and 52 h **(g–i)** after the start of the organ culture. Note that 70% of KO lungs present a phenotype (KO+) and 30% are comparable with WT (KO–). Scale bar, 0.5 mm.

As a measure for branching, the number of terminal endbuds was counted on micrographs as shown in Figure 1. The growth (volume%) was estimated by area measurement of the projected organ pieces by point counting. The growth was calculated by dividing the measured area by the area of the explant at the start of the experiment and multiplying this result by 100. We may take these numbers as an estimation of the volume percentage of the growth, because the quotient of the diameter and the thickness of an individual explant was constant, especially as compared between different explants at one time point, but also between explants at different time points (Schittny et al., 2000; Roth-Kleiner et al., 2004). The α8-deficient lung explants were grouped according to their difference to WT explants. If after 48 h in culture the difference between α8-deficient and WT explants was less than 15%, the explants were defined as α8-deficient explants without phenotype (KO−) all other α8-deficient explants were defined as showing a phenotype (KO+).

### Morphology

Lungs were prepared as previously published (Luyet et al., 2002; Mund et al., 2008; Tschanz et al., 2014). Briefly, at postnatal days P4, P7, P10, and P90 the pulmonary blood vessels of five animals per group and day were perfused with phosphate buffered saline (PBS = 10 mM sodium phosphate, containing 127 mM sodium chloride, pH 7.4), containing 5 units/ml heparin, 10 mg/ml procaine and 10 mM EDTA (Fluka Chemie AG, Buchs, Switzerland), and the air space filled with PBS, containing 4% freshly prepared paraformaldehyde (Merck, Darmstadt, Germany) at a constant pressure of 20 cm water column. At this pressure, the lung reaches roughly its total lung capacity (TLC). In order to prevent a recoiling of the lung, the pressure was maintained during fixation of 1 h. After fixation, the five lobes were separated and their volumes were measured by water displacement (Scherle, 1970). The total lung volume was calculated from the five lobar volumes. The specific lung volume was defined as lung volume over body weight (Weibel, 1970). Lungs were embedded in paraffin (Histosec, Merck, Darmstadt, Germany) at 60°C after three washes in PBS, followed by a graded series of ethanol and by three changes of Histoclear (Life Science International, Frankfurt, Germany). Under all conditions no recoil of the lungs was observed. A total of 3.5–5 μm sections were cut, transferred onto silanized (aminopropyl-trimethoxy-silane) micro slides, air dried over night at 37°C, dewaxed in Histoclear and stained with hematoxylin-eosin.

### Stereology

Stereological measurements were done as previously published by our group (Mund et al., 2008; Schittny et al., 2008; Tschanz et al., 2014) and in compliance with standards for quantitative assessment of lung structure of the American Thoracic Society and the European Respiratory Society (Hsia et al., 2010). Briefly, 40–50 images per animal were taken at a magnification of ×250 on 10–12 sections of its left lung. Sampling of the sections and the images were done according to a systematic random sampling scheme (Cruz-Orive and Weibel, 1981). The sections were taken at equal distances to each other covering the entire length of the lung. The start of this series of sections was determent randomly. Five left lungs per day and group were analyzed. It was shown that the left lung represents a valid sample for the entire lung (Zeltner et al., 1990; Barre et al., 2016). The volume of the five lobes was measured by water displacement (Scherle, 1970) (see above). Volume densities were estimated by point counting. The lung parenchyma was defined as airspaces and septal tissue, excluding bronchi, bronchioli, and blood vessels >20 μm in diameter. Intersection counting was used to estimate the septal (alveolar) surface area density. The length density of the free septal edges was estimated by counting the number of cut free septal edges (tips of the cut septa) in a reference area on 2D paraffin sections. The total length of the free septal edge represents the total length of all alveolar entrance rings. (Mund et al., 2008; Schittny et al., 2008; Roth-Kleiner et al., 2014; Mund and Schittny, 2020). The totals of the parenchymal volume, the septal surface area, and the length of the free septal edge were calculated by multiplying their densities by the total parenchymal lung volume (Weibel, 1984; Howard and Reed, 2005). All calculations were done separately for each animal and each time point. In order to calculate the extent of newly formed alveolar septa, the length density was mathematically corrected for its change due to the growth of the volume of the lung parenchyma. An enlargement of the lung without the addition of new septa will lead to a decrease of the length density of the free edges by a factor of ^3^√(*V*_x_/*V*_0_)^2^ (where *V*_x_ represents the parenchymal lung volume at the time point X and *V*_0_ the volume at the start of the growth). We use this correction because volume increases by a factor of *x*^3^ and length only by a factor of *x*^1^. We corrected the stereologically estimated length densities for its decrease due to isometric growth of the lung parenchyma by multiplying by the factor of ^3^√(*V*_x_/*V*_0_)^2^ (thereby *V*_0_ was set to the mean value of the parenchymal lung volume at postnatal day 4). During isometric growth of the lung the resulting “growth corrected length density” remains constant and shows an increase or a decrease, if new septa are formed or if septa are degraded, respectively. We calculated the formation of new alveolar septa during lung development and compared it between controls and α8-deficient mice by following the increase of the “growth corrected length densities” between postnatal days 4 and 90. In Tschanz et al. (2014) we discuss the method described above in more details and compared the number of alveoli with the length of the free septal edge throughout lung development.

### Statistical Analysis

Statistical analysis was done using 2-way ANOVA and Bonferroni post-test. Significance was defined as *P* < 0.05 and “highly significant” as *P* < 0.001 (Altman, 1991). Microsoft Excel (Office 2010, Microsoft Corporation, Redmond, WA, United States) and Prism (version 5.04, GraphPad Software Inc.) were used to calculate the statistics.

## Results

### Integrin α8-Deficiency Reduces Branching Morphogenesis

To assess the role of the integrin α8 subunit during branching morphogenesis, we collect explants from wild type (WT) and α8 deficient mice (KO) at embryonic day E11.5–E12.5 and cultured them for up to 3 days (Table 1). Explants were imaged twice a day (Figure 1). Day E11.5 (embryonic stage) represented the earliest day, where we could start the lung organ culture. We included day E12.5 (pseudoglandular stage) in order to extend the time interval of observation. We did not observe any significant difference between days E11.5 and E12.5 (data of E12.5 not shown).

**FIGURE 2:**
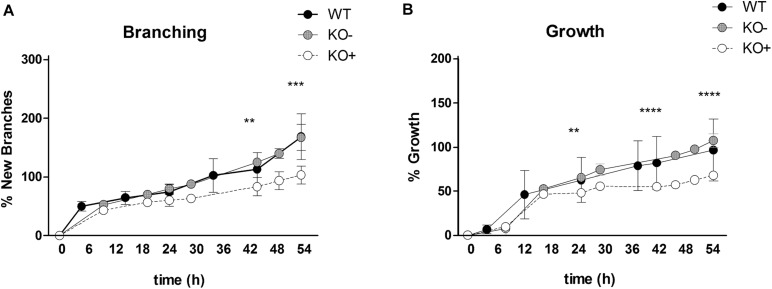
Quantification of new branch formation and lung growth. The percentage of new branching was assessed by counting the terminal endbuds at indicated time points from E11.5 explants **(A)**. The percentage of the lung growth was estimated by measuring the area of the projected organ by point counting **(B)**. The lung explants were clustered into three groups: wild type (black, WT), α8 deficient explants without phenotype (gray circles, KO–), α8 deficient explants with phenotype (open circles, KO+). Data were analyzed by 2-way ANOVA and Bonferroni post-test, **P = 0.01, ***P = 0.001, and ****P = 0.0001.

At time 0, WT and KO explants appeared comparable (Figures 1a–c). After 30 h of organ culture most, but not all of the KO explants showed a small reduction of branches as compared to WT explants (Figures 1d–f). A marked reduction of airway branching was evident in KO explants after 43 h of organ culture (Figures 1g–i, shown for 52 h). The deficiency, as verified by quantification, consists of diverse defects in branching and bud formation. Interestingly, only ∼70% of the KO explants (Figure 1h) showed a pronounced phenotype (KO+) with reduced branching, the remaining ∼30% (Figure 1i) showed the same number of branches (KO−) as WT (Figure 1g). New branching formation was quantified for each explant at different time points and significant differences were detected between WT and KO+ explants after 43 h of lung culture. No difference was observed between WT and KO− (Figure 2A). In addition, we asked whether α8 deficiency has a direct effect on the lung growth. Lung size was estimated by measuring the area of the projected organ at indicated time points and statistical differences were observed between WT and KO+ groups especially after 24, 42, and 54 h of organ culture (Figure 2B). These data suggest that loss of integrin α8β1 alters not only the branching morphogenesis but also the overall growth of the lung explant.

### Integrin α8-Deficiency Shows Altered Branching Patterns

**FIGURE 3:**
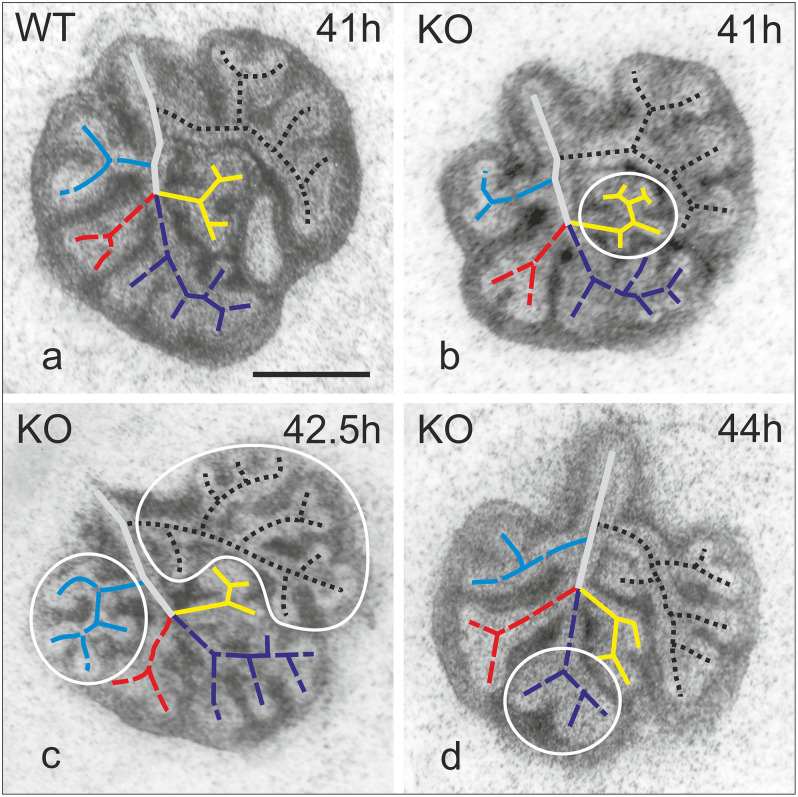
Map of branching patterns in WT and α8KO explant cultures of E11.5 lungs. Representative WT lung **(a)**. KO lungs regardless of the phenotype (KO+ or KO–) 41–44 h after the explant **(b–d)**. Irregularities are encircled. Black dotted line indicates branching in the left lung; in blue, cranial lobe of right lung; in red, middle lobe; purple caudal lobe; yellow, accessory lobe. Bar 0.5 mm.

Experiments were performed to evaluate whether the lack of α8 integrin affects branching pattern during pulmonary development. Lungs from WT and KO obtained at E11.5–E12.5 were cultured and imaged at different time points (Figure 3). All WT lungs showed a normal pattern of branching over time (Figure 3a). Deficiency of α8, regardless of the penetrance of the phenotype caused an altered branching pattern (Figures 3b–d). Abnormal branching patterns were observed inside the lobes and interestingly, none of the fives lobes was more affected than the others (Figures 3b–d irregularities are encircle).

### Integrin α8-Deficiency Affects Lung Spontaneous Contraction and Bud Morphology

**FIGURE 4:**
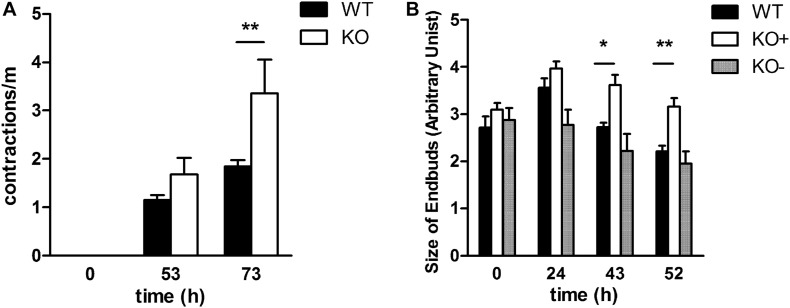
Spontaneous contractions and size of terminal buds in E11.5 explant cultures over time. **(A)**. Contractions were measured in WT and KO explants at starting point E11.5 and after 53 and 73 h of organ culture **(B)** The size of the endbuds was determined by measuring projected surface area at the start of the culture of WT, KO–, KO+ E11.5 (time 0) explants, as well as after 24, 43, and 52 h of cultivation. Data were analyzed by 2-way ANOVA and Bonferroni post-test, *P = 0.05 and **P = 0.01

It is known that contractions of the future airways contribute to a normal airway differentiation and branching (Schittny et al., 2000) and α8β1 is expressed in contractile interstitial cells (CICs) (Levine et al., 2000). To determine whether the lack of α8 affects this ability to contract, we analyzed and counted the number of contractions per minutes after 53 and 73 h in culture of E11.5 and E12.5 explants (Figure 4A). We found that contractions increased with time in WT and KO independently of the presence of the phenotype. Interestingly KO showed a significantly higher frequency of contractions after 73 h of organ culture. Furthermore, we studied whether the size of terminal endbuds was affected by α8-deficiency. Photographs taken at the day of explant (0) and after 24, 43, and 52 h in culture were quantified (Figure 4B). The size of endbuds was similar between WT and KO−, but interestingly KO+ showed larger endbuds than WT.

### Anatomical Differences Between WT and α8 Deficient Mice

**FIGURE 5:**
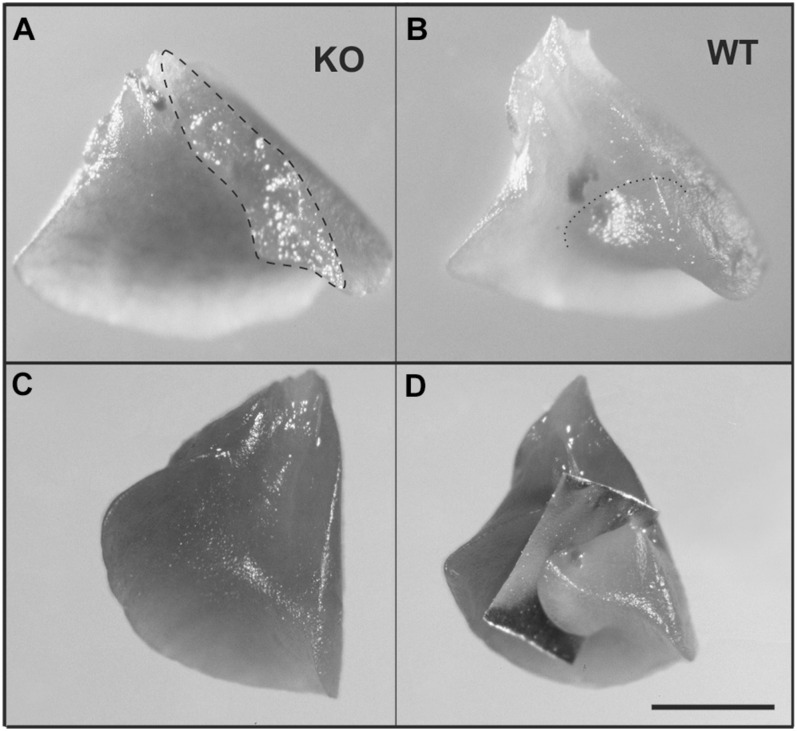
Anomalous lung lobation in KO young adult as compared to WT. **(A)** Caudal lobe from KO lung with incomplete separation between the middle and the caudal lobe of the right lung. **(B)** Caudal lobe from WT. **(C)** KO caudal lobe from KO with incomplete or missing fissure **(C)** which is present in WT lungs (**D**, an aluminum foil demonstrates where the fissure is running). Dashed line represents area of incomplete separation **(A)**; dotted line indicates fissure in WT **(B)**. Scale bar is 2 mm.

The pulmonary phenotype of α8-deficient mice is characterized by a fusion of the medial and caudal lobe, as a consequence of mesenchymal and mesothelial cell anomalies and defective saccular airway branching (Benjamin et al., 2009). We found the same phenotype as Benjamin et al. (Benjamin et al., 2009) (Figure 5A) in the KO lungs where fusion is evident when compared to WT (Figure 5B). In addition, WT lungs clearly presented a fissure, which was completely missing in KO lungs (Figures 5C,D).

### Integrin α8 Deficiency Induces Prominent Formation of New Septa With a Consequent Rescue of the Phenotype

**FIGURE 6:**
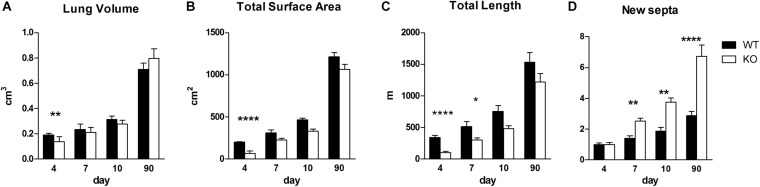
Stereological measurements in WT and KO postnatal lungs. Stereological analysis of lung volume (cm^3^) **(A)**, total surface area (cm^2^) **(B)**, total length of the free septal edge (m) **(C)** and formation of new septa (expressed as multiples of the septa present at day 4) **(D)** in lungs of WT and KO mice at indicated time points. Data were analyzed by 2-way ANOVA and Bonferroni post-test, *P < 0.05, **P < 0.01, and ****P < 0.0001; N per group, 5.

**FIGURE 7:**
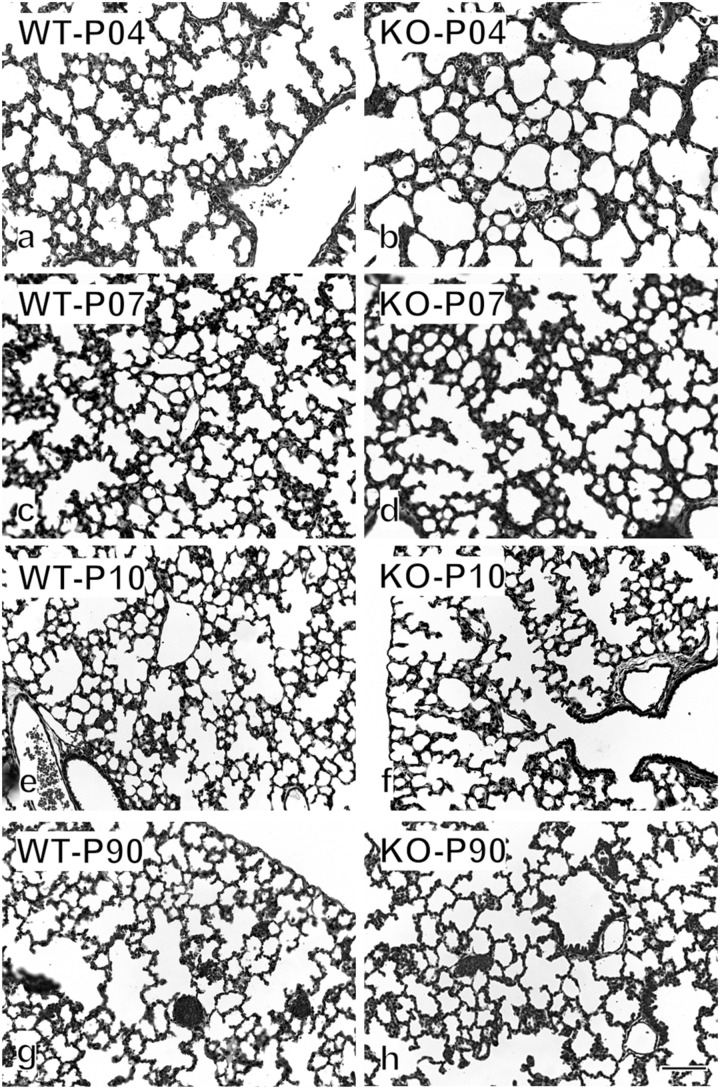
Histological appearance of WT and KO lung throughout lung development. Examples of the original images used for the stereological estimations are shown on postnatal days 4 **(a,b)**, 7 **(c,d)**, 10 **(e,f)**, and 90 **(g,h)**. Already by eye, a significant difference is visible between WT and KO on P4. Later the difference disappears gradually. Bar, 100 μm.

We asked whether the phenotype observed in KO organ cultures could lead to a characteristic phenotype postnatally (Figures 6, 7). We investigated the impact of α8 integrin deficiency during alveolarization. Lung sections were compared between WT and KO littermates at postnatal day 4, 7, 10, and 90. Absolute lung volume and total surface area was significantly reduced in KO lungs as compared to age-matched WT at postnatal day 4 (P4) (Figures 6A,B).

During the phase of alveolarization, the increase of structural complexity of the lung parenchyma may be view from two different points of view: we look either to the formation of new alveoli or to the formation of new alveolar septa. Both parameters are well suited to study postnatal rodent lung development (for a comparison see Schittny et al., 2008; Tschanz et al., 2014). We focused on the formation of new alveolar septa and estimated the total length of the free septal edge. This parameter represent the total length of all septa, measured at their upper free (not connected to anything) edge. In a first approximation, it is equal to the total length of the alveolar entrance rings. A significant reduction of this parameter was detected in KO lungs at P4 and P7 (Figure 6C). Interestingly, we found that the phenotype observed at an early stage, P4, did not persist until adulthood (P90): lung volume, total surface area and total length of free septal edge were comparable between WT and KO lungs (Figures 6A–C). These findings may be explained by the results of the stereological estimations showing that a significantly larger amount of new septa is formed in KO animals as compared to WT between postnatal days P4 and P90 (Figure 6D). In all KO lungs we observed the same postnatal phenotype and rescue. Therefore, postnatally the penetration of the phenotype was complete.

## Discussion

### Phenotypes and Its Penetration

In the present study, we demonstrate that integrin α8β1 is essential for the proper development of the lung and its absence causes many defects during prenatal and postnatal development. During prenatal lung development of α8 KO mice, defects include reduced and irregular branching morphogenesis, a higher frequency of spontaneous contractions of the future airways and anomalous lobation. However, in the α8 KO animals, we observed only ∼70% of penetrance of the prenatal branching phenotype. Similar results were found previously (Muller et al., 1997) in the kidney of α8 KO mice, which are born with different kidney phenotypes. The presence of variable phenotypes is partially explain as a consequence of the two genetic background (129 and C57Bl/6) present in those mice. It is possible that the presence or the absence of genetic modifier locus controls specifics phenotypes. We assume that not only the two genetic backgrounds modulate the penetrance but also a compensatory effect by other integrins during the different steps in lung development. Based on kidney studies it has been hypothesized that α5 and αV could compensate the function of α8, but it was not the case due to the negative results obtained (Haas et al., 2003). Moreover, collagens I and III were detected in the glomeruli of α8 KO mice leading to the assumption that compensation for α8 function could implicate interactions between collagens with collagen receptors. We speculate that similar mechanisms involved in the renal phenotypes are also responsible for the lung phenotype, which we observe.

During postnatal development, an increased formation of new septa was observed resulting in a nearly normal lung in adult KO mice (day 90). In opposite to the prenatal phenotype we observed a complete penetration of the postnatal phenotype. Previous studies (Wagner et al., 2003; Benjamin et al., 2009) have shown cellular function and spatiotemporal expression of α8β1 in the developing lung. However, the specific prenatal and postnatal defects, which were observed in this study, have not been described until now.

### Branching Morphogenesis

Branching morphogenesis is a complex phenomenon regulated by signaling factors that requires epithelial and mesenchymal interactions to form an epithelial bud (Iber and Menshykau, 2013). Only rare variations are seen in lungs of WT mice proving that branching is not random but a very well controlled mechanism (Metzger and Krasnow, 1999). We observed that α8 KO lungs with a phenotype (KO+) showed fewer branches and reduced growth compared to WT and KO without phenotype (KO−, Figures 1, 2) in lung organ cultures. In addition, all KO lung explants showed an irregularly altered branching pattern (Figure 3). Any culture system has it limits if it is compared to a live animal. However, the phenotype detected in lung organ culture was confirmed by the observation that at postnatal day 4 the lung volume, the total alveolar surface area, and the total length of the free septal edge were reduced in live animals. These results represent a confirmation, because at this time point alveolarization did not start yet (see section “Alveolarization” and Figures 6A–C).

Interestingly, former studies have shown that tenascin-c (TNC) represents a ligand for α8 integrin (Schnapp et al., 1995b). In addition, previous research in our laboratory (Roth-Kleiner et al., 2004) has demonstrated that TNC deficiency leads to a reduction of branching but showed normal overall growth of the explants and normal branching patterns in deficient mice. Branching and branching patterns are governed by a dynamic process of epithelial–mesenchymal interactions (Morrisey et al., 2013). Because tenascin-C as well as α8 integrin deficiency shows similar phenotypes, it may be concluded that both are involved in the epithelial–mesenchymal interactions during lung development. Typically α8 integrin and TNC-mRNA is expressed by mesenchymal cells, but the TNC-protein was found along the epithelial basement membranes (Zhao and Young, 1995). Therefore, we hypothesized that TNC signals to the epithelial and mesenchymal cells and influences their adherence to their substratum (Chiquet-Ehrismann and Tucker, 2011) and that α8 recognizes TNC (Schnapp et al., 1995b) and closes at least one loop of interactions.

### Spontaneous Contractions of the Future Airways

During gestation, spontaneous contractions of the future airways have been described (Schittny et al., 2000). The responsible players of those contractions are airway smooth muscles. They contribute to normal lung development and fetal lung growth (Schittny et al., 2000). Murine and rat lung development studies have shown that primitive lung mesenchymal cells retain a generic capacity to differentiate into airway smooth muscle cells, and these cells follow the emergence of the definitive lung bud (Jesudason et al., 2005). In our study, we also noticed spontaneous contractions of the future airways in WT, KO−, and KO+. Independently from the phenotype, KO lungs showed a significantly increased number of contractions per minute (Figure 4A) and this correlates with an increase of the size of the endbuds (Figure 4B). We propose that absence of the α8 subunit could lead to signals, which activate myofibroblasts or reduce an inhibitor pathway with the result of faster contractions.

### Alveolarization

Alveolarization is a complex process coordinated by multiple interactions between extracellular matrix, fibroblasts, epithelial cells, and microvasculature. Deficiencies in one of these elements have consequences on the entire alveolar development. Interestingly, at postnatal day 4, α8 deficient mice showed a significant reduction in lung volume compared with WT (Figure 6). Because at day 4, alveolarization did not yet start, this phenotype is exclusively caused by branching morphogenesis. However, at day 90 KO lungs were comparable to WT (Figures 6, 7). Therefore when mice are considered to be adults WT and KO do not show any significant difference. The same holds true for the total surface and total free septal length. The latter represents a measure for alveolarization. Based on morphological measurements we found that KO mice start with one fourth of length of the free septal edge at P4 (Figure 6C) and in order to become equal to WT, KO mice compensate with a massive input of new septa (Figure 6D). Therefore, the prenatal phenotype of the α8 deficient mice is rescued by an increased alveolarization during postnatal life.

It may be speculated about the size of the acini during the rescue of the α8-phenotype between days 4 and 90. It is likely that in the KO+ mice branching is as well reduces as the number of acini. Because the number of acini is most likely constant in postnatal mouse lung development (Barre et al., 2014, 2016), we hypothesize that KO+ mice possess a smaller number of acini which show a larger volume as compared to wildtype mice. A similar – at the end unanswered question – arise during compensatory growth after pneumonectomy: The remaining lung is able to regrow to its original size and alveolar surface (Hsia et al., 1994; Hsia and Johnson, 2006; Butler et al., 2012). If our data, that the number of acini stays constant after the saccular stage (Barre et al., 2016), are right, the only other known option will be to increase the size of the acini.

Tenascin-C is not only contributing to branching morphogenesis, but also to alveolarization. It involved in hyperoxia- (Olave et al., 2016) and steroid-induced (Roth-Kleiner et al., 2014) abnormal lung development. Furthermore, tenascin-C deficient mice show in both phases of alveolarization (classic and continued) a deceleration and acceleration of alveolarization (Mund et al., 2008; Mund and Schittny, 2020). Therefore, even if tenascin-C is a ligand of α8 integrins they exhibit different phenotypes during pulmonary alveolarization.

### Conclusion

Taken together our results indicate that α8β1 integrin plays several roles in distinct phases of lung development. During prenatal lung development, regular spatio-temporal presence of α8β1 is essential for the normal pattern of branching. Most likely α8 has a direct effect on branching and on the branching pattern, but in addition an indirect effect by the alteration of the spontaneous contractions of the future airways. From these observations, we conclude that α8β1 is crucial for lung development at different time points even though the mechanism responsible for the rescue of the lung phenotype is still unknown. But the fact that a rescue takes place during alveolarization shows the enormous potential of the lung to correct early impairments of lung development. We assume that during the development chronic disease (such as emphysema, COPD and fibrosis) the lung is overwhelm by inflammation and activation of specific pathways, which do not consent the correction of septa loss.

Although numerous elements in the process of alveolarization have been determined, more studies are needed in order to clarify the complex interrelationships that are operative during normal lung development.

Our findings strongly suggest the necessity of further studies in order to determine how to improve the mechanism able to correct impairment in the lung and which is the point of no return when this mechanism is not anymore able to correct it.

## Data Availability Statement

The datasets generated for this study are available on request to the corresponding author.

## Ethics Statement

The animal study was reviewed and approved by Veterinary Service of the Canton of Bern and the Canton of Basel, Switzerland and the Swiss Federal Agency for Environment, Forest and Landscape.

## Author Contributions

TC analyzed the data and drafted the manuscript. AH obtained the postnatal lungs and did the required breeding of the mice. JS conceived and designed the study, performed the lung organ cultures including the videotaping and analysis of the data. In addition, he did of parts of the breading of the mice, the stereological estimations of postnatal lung development including analysis of data, and contributed to writing. All authors contributed to the article and approved the submitted version.

## Conflict of Interest

The authors declare that the research was conducted in the absence of any commercial or financial relationships that could be construed as a potential conflict of interest.

## Abbreviations

α8: α8 integrin subunit
E: embryonal
P: postnatal
WT: wild type
KO: α8 deficient mice or lungs
KO+: α8 deficient mice or lungs with phenotype
KO−: α8 deficient mice or lungs without phenotype.
